# Design and development of a disease-specific clinical database system to increase the availability of hospital data in China

**DOI:** 10.1007/s13755-023-00211-4

**Published:** 2023-01-30

**Authors:** Mimi Liu, Jinni Luo, Lin Li, Xuemei Pan, Shuyan Tan, Weidong Ji, Hongzheng Zhang, Shengsheng Tang, Jingjing Liu, Bin Wu, Zebin Chen, Xiaoying Wu, Yi Zhou

**Affiliations:** 1 Zhongshan School of Medicine, Sun Yat-Sen University, Guangzhou, Guangdong China; 2 Gastroenterology Department, The Third Affiliated Hospital of Sun Yat-Sen University, Guangzhou, Guangdong China; 3 Center of Hepato-Pancreatico-Biliary Surgery, The First Affiliated Hospital, Sun Yat-Sen University, Guangzhou, Guangdong China

**Keywords:** Disease-specific clinical database system, Medical big data, Clinical data repository, Clinical database, Liver cirrhosis, Disease dataset, Clinical data aggregation

## Abstract

**Purpose:**

In order to meet restrictions and difficulties in the development of hospital medical informatization and clinical databases in China, in this study, a disease-specific clinical database system (DSCDS) was designed and built. It provides support for the full utilization of real world medical big data in clinical research and medical services for specific diseases.

**Methods:**

The development of DSCDS involved (1) requirements analysis on precision medicine, medical big data, and clinical research; (2) design schematics and basic architecture; (3) standard datasets of specific diseases consisting of common data elements (CDEs); (4) collection and aggregation of specific disease data scattered in various medical business systems of the hospital; (5) governance and quality improvement of specific disease data; (6) data storage and computing; and (7) design of data application modules.

**Results:**

A DSCDS for liver cirrhosis was created in the gastrointestinal department of a 3A grade hospital in China and had more than nine data application modules. Based on this DSCDS, a series of clinical studies are being carried out, such as retrospective or prospective cohorts, prognostic studies using multimodal data, and follow-up studies.

**Conclusion:**

The development of the DSCDS for liver cirrhosis in this paper provides experience and reference for the design and development of DSCDSs for other specific diseases in China; it can even expand to the development of DSCDSs in other countries if they have the demand for DSCDS and the same or better medical informatization foundation. DSCDS has more accurate, standard, comprehensive, multimodal and usable data of specific diseases than the general clinical database system and clinical data repository (CDR) and provides a credible data foundation for medical research, clinical decision-making and improving the medical service quality of specific diseases.

**Supplementary Information:**

The online version contains supplementary material available at 10.1007/s13755-023-00211-4.

## Introduction

In China, medical informatization started relatively late, and it was not until the early 1990s that hospitals began to establish information systems, mainly being hospital information systems (HIS). Their functions were for hospital management and operations, such as writing electronic medical records, issuing medical orders and charging medical fees. Subsequently, hospitals gradually began to establish picture archiving and communication systems (PACS), laboratory information management systems (LIS), electronic medical records (EMR), radiology information systems (RIS), hospital resource planning systems (HRP), nursing systems, etc. [1].

However, hospitals established these systems primarily to achieve specific medical business and management goals and to improve the quality and efficiency of medical services with information technology to a certain extent. Therefore, these systems are collectively referred to as hospital business systems. At the same time, hospitals did not have unified planning and implementation of these business systems but chose hospital information technology (HIT) companies and designed data application modules of these business systems according to their medical informatization needs in different periods, length of the construction period, amount of construction funds, etc. The patients’ medical information was stored in various hospital business systems, and the data standards were not unified. There were even many “information islands” among these business systems, so it was impossible to realize unified information queries of a specific disease or a patient, and information extraction from these systems was also very complex and cumbersome [1, 2].

The above situation and problems are common in the development of hospital medical informatization in China. Therefore, these hospital business systems store a large amount of real world medical data. These data have great potential value, but they are difficult to mine and utilize due to the lack of research-based database systems oriented to solving the clinical problems of diseases. To fully leverage the value of real world medical data, the hospitals began to govern the data stored dispersedly in various hospital business systems in China [3]. They established clinical data repositories (CDRs) and general clinical database systems to provide high-quality data for clinical research, including improving medical service quality, predicting treatment effects, reducing medical risks, and controlling the medical costs of certain diseases [4, 5]. Based on the above, the disease-specific clinical database system (DSCDS) was gradually emerging. DSCDS was defined as a clinical database system that focused on a specific disease and contained multimodal and multisource heterogeneous clinical data. These data could be efficiently used for clinical research, medical diagnosis and treatment of the specific disease. However, until recently, the hospitals have just started to construct DSCDSs for a few specific diseases, including bone tumors, arteriosclerosis obliterans of the lower limbs, pituitary diseases, thyroid tumors, breast cancer, thymoma, ovarian cancer, ankylosing spondylitis, renal cancer, Parkinson's disease, etc. However, there is no relatively clear, standard and systematic design and implementation scheme of these DSCDSs and no DSCDS for liver cirrhosis, which greatly limits the scalability and application range of these DSCDSs.

In this study, a DSCDS was designed and built for liver cirrhosis in a 3A grade hospital that is good for diagnosing and treating liver diseases. This DSCDS met the gastroenterologists’ needs for specialized data of liver cirrhosis, connected the “information islands” and shared the specialized disease information on liver cirrhosis in the whole hospital. Moreover, it has provided strong data support for comprehensively utilizing multimodal data (such as structured data, text, images and clinical biological samples, etc.) to carry out clinical research and apply to precision medicine for liver cirrhosis, which was helpful to further improve the clinical standardization and levels of diagnosis and treatment for liver cirrhosis. Meanwhile, for this study, we also hoped to provide a referential design and implementation scheme for other DSCDSs for more specific diseases in China and even in other countries.

## Methods

### Requirement analysis

#### Needs for developing precision medicine

The occurrence and development of disease is a long-term and complex process, and various factors affect the course of the disease, bringing about considerable individual differences among patients even with the same disease. Thus, patients with a particular disease need more precise clinical diagnosis, treatment and health care, which is the reason for developing precision medicine. With the development of precision medicine, clinical research pays increasing attention to specific diseases, especially in the research and decision-making of some complex diseases [6]. These types of sophisticated and in-depth clinical research and decision-making need to analyze and mine more comprehensive, accurate, and massive data on specific diseases [7–9]. Therefore, it is necessary to collect specific disease data scattered in various hospital business systems to establish DSCDS and fully tap the knowledge contained in medical big data in DSCDS to provide reliable and precise evidence for clinical research and decision-making [10–12].

#### Real world medical big data need to be aggregated and utilized urgently

China has a large population base, accounting for approximately one-fifth of the world's population. Thus, the cumulative number of patients with various specific diseases treated in hospitals is significant, as well as the data of these specific diseases, particularly in some hospitals that specialize in diagnosing and treating several specific diseases. A large amount of medical data can continuously provide a stable data source for constructing DSCDSs and large-scale clinical research [8]. However, it has not yet been truly aggregated and utilized in hospital business systems. Therefore, in China, while spending extra resources to perform cohort studies to collect specific disease data for clinical research may not be necessary, establishing DSCDS based on the existing hospital business systems is urgent, so that real world clinical medical data can be used to support many clinical studies on specific diseases [13, 14], and some unnecessary and repetitive investment of financial and human resources can also be reduced in clinical research.

#### Needs of clinical research on specific diseases

Some specific disease databases have been constructed gradually in China since the beginning of the twenty-first century, but they were generally small datasets and built to carry out specific research. These databases had relatively single data sources, poor data sharing, and uneven data quality; some databases had irregular data formats and inconsistent information standards due to manual data entry and processing. As a result, these databases had low data utilization, which brought inefficient application and transformation of research results. In addition, they were not DSCDSs, which are defined in this paper, nor could they meet the increasing needs of clinical research on specific diseases.

In addition, some hospitals have also begun to build CDRs to gather medical data from the whole hospital to further improve the efficiency of hospital management and medical services. However, the primary purpose of developing these universal CDRs was to ensure data integrity, that is, to obtain comprehensive medical data that was inclusive of most hospital business systems and all diseases. A relatively single standard was adopted to aggregate data of multiple diseases. Therefore, CDRs could neither precisely extract different information about the clinical manifestations and signs of different diseases nor meet the requirements of clinical research for more comprehensive data sources on specific diseases [15]. Therefore, it is urgent to establish DSCDS, which realizes the individualized design of a clinical database system based on a specific disease, and use big medical data in DSCDS through emerging technologies (such as those involving data analysis and mining, artificial intelligence, etc.) to master the characteristics of occurrence, development, and prognosis of the specific disease with different etiologies and to finally clarify the pathogenesis of the specific disease to assist clinical decision-making as finalization. All of these factors have clinical importance for delaying the progression of specific diseases, accurately predicting the occurrence of complications and the risk of death, and early implementation of treatment and intervention for patients with specific diseases. The above requirement analysis of DSCDS is also applicable to other DSCDSs for most specific diseases, including liver cirrhosis.

### Design schematics and basic architecture

**Fig. 1:**
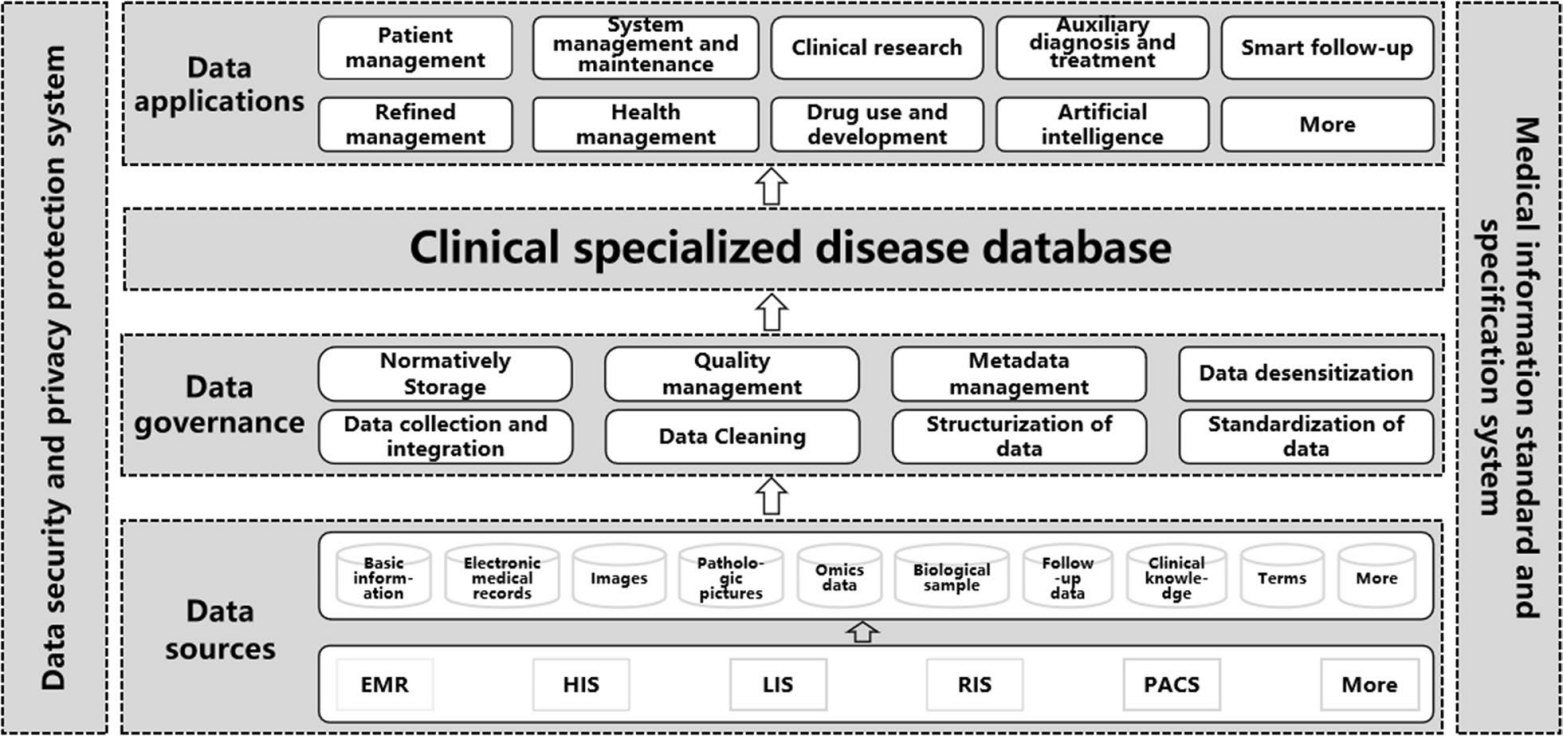
The design schematics and basic architecture of DSCDS. *EMR* electronic medical record, *HIS* hospital information system, *LIS* laboratory information management system, *RIS* radiology information system, *PACS* picture archiving and communication system

A distributed architecture was adopted to normatively collect specific disease data from various sources (such as Oracle, SQL Server, MySQL, NoSQL, etc.), integrate the data stored in hospital business systems (such as HIS, PACS, LIS, EMR, RIS, etc.) into a database using standard data models and realize the information integration of patients with the same specific disease from different departments, information systems and modal data in hospitals. Next, some algorithm engines (such as natural language processing, knowledge mapping, machine learning, deep learning, artificial intelligence, etc.) were used to govern the data, including extraction, transformation and loading (ETL), cleaning, structuralizing, standardizing, and normalizing the data; then, indicators were selected from specific disease datasets through feature extraction to establish a standardized database system that was a DSCDS. DSCDS provided more than nine data application modules for medical services and clinical research on specific diseases. The design schematics and basic architecture of DSCDS are depicted in Fig. 1.

In addition, in each period of the construction of DSCDS, a data security and privacy protection system was followed, including access control, authentication, access security, application security, data security, network security, system environment security, physical security, emergency handling, disaster recovery plans, and security technologies (such as desensitization and encryption). They were all derived from China's and even international policies, regulations and standards on medical information security and privacy protection. At the same time, a medical information standard and specification system was also referenced, including data standards, exchange standards, service standards, management standards, security standards, technical standards, etc.

### Standard dataset and data model

The difficulties in the construction of DSCDS lie in how to integrate multisource heterogeneous data. It is necessary to define a set of common data elements (CDEs) that enable the systematic collection, analysis and sharing of data and to build standard data models to gather more multimodal data from additional data sources to establish standard datasets of specific diseases [2]. This paper refers to and expands on the existing data elements from some international standard medical datasets and medical terminologies from many international clinical guidelines, authoritative expert consensus, high-quality academic papers, medical textbooks, and monographs related to specific diseases and considers the actual situation of the diagnosis and treatment of specific diseases in China. Finally, a set of CDEs of DSCDS is defined, and the CDEs of DSCDS have a group of properties such as code, first classification, secondary classification, name, definition, data type, data presentation format, value range, source of standardization, and memo.

The CDEs of DSCDS included two parts: the public data elements of most diseases (PDEs) and the specialized data elements of a specific disease (SDEs). The former involved multidimensional information such as hospitalized information, demographic information, health history, chief complaint and symptom, physical examination, laboratory examination, clinical assistant examination, diagnosis, medical assessment, medical planning and intervention, clinical biological samples, medical expenditure, and medical institution, with a total of approximately 350 data elements. The latter had more data elements expanded from the above 13-dimensional information of PDEs according to the needs of clinical research on the specific disease. Therefore, the total number of SDEs varies depending on the specific disease. Generally, the number of CDEs of DSCDS is approximately 450–1000 (see Table 1 and details in the Online Appendix).

**Table 1:** The first and secondary classification of CDEs of DSCDS

First classification	Secondary classification
Clinical visit information	Personal identification; clinical visit identification; clinical visit records
Demographic information	Personal information; address; contact information
Health history	Past medical history; surgical history; smoking history; history of alcohol consumption; reproductive history (female); menstrual history (female); familial disease history; allergy history; more life histories linked to a specific disease (e.g., exposure to pathogenic factors, blood transfusion, drug abuse, tattoo)
Chief complaint and symptom	Chief complaint; symptoms (symptoms vary widely among specific diseases, many of them are common symptoms of specific diseases, such as weight change, abdominal pain, fatigue, fever, anorexia, abdominal distention, diarrhea, hemoptysis, hematemesis, melena, anemia, abdominal mass, etc.)
Physical examination	General examination; vital signs; score; more physical examinations linked to a specific disease (e.g., state of consciousness, nutritional status, abdominal tenderness, perianal pathological changes)
Laboratory examination	Blood routine examination; routine urine examination; blood biochemical examination; Hepatitis B test; More infectious disease examinations linked to a specific disease (e.g., hepatitis A, hepatitis C, hepatitis D, hepatitis E, syphilis); feces routine examination; Routine examination of hemorrhage and coagulation; more laboratory tests linked to a specific disease (e.g., tumor markers detection, sex hormone detection, disease-specific antigens detection, gene test, liver function test, determination of liver fibrosis, kidney function tests)
Clinical assistant examination	Imaging examination (including X-ray, CT, magnetic resonance, ultrasound, nuclear medicine, etc.); more clinical assistant examinations linked to a specific disease (e.g., immunohistochemistry, pathological examination, pathological biopsy, endoscopy)
Diagnosis	Clinical diagnosis; medical diagnosis on death; more medical diagnoses linked to a specific disease (e.g., stage of disease, first diagnosis of tumor, complication); more comorbidities linked to a specific disease (e.g., malignant tumor, kidney disease, diabetes, hypertension, heart disease, cerebrovascular disease)
Medical assessment	Grouping of risk; relapse; more medical evaluation linked to a specific disease (e.g., tumor metastases, etc.)
Medical planning and intervention	Hospitalization information; drug therapy; clinical trials; surgery; postoperative recovery; more operations linked to a specific disease (e.g., needle biopsy for malignant tumor); more treatments linked to a specific disease (e.g., antiviral therapy, radiotherapy, and symptomatic treatment and endoscopic therapy); treatment evaluation; follow-up information
Clinical biological samples	Serum; feces; more clinical biological samples linked to a specific disease (e.g. tissue, saliva, urine, bone marrow)
Medical expenditure	Medical expenditure
Medical institution	Institution identification

Notes: The details for the PDEs and SDEs of DSCDS in the appendix, and the SDEs of liver cirrhosis were taken as examples.

### Collection and aggregation of multisource heterogeneous data

To combine DSCDS with as much clinical information as possible and to integrate the bottom data of hospital business systems, database synchronization technology and ETL technology were applied to collect and aggregate multisource heterogeneous data of a specific disease.

The following key processes were developed: (1) data storage and access based on standard RESTful protocol, standard XML/JSON format, and standard messaging protocol; (2) customizing the type of data source and the fields that need to be mapped to intelligently fit heterogeneous data sources, customize data processing, and flexibly implement data processing strategies; (3) collecting and aggregating data in real-time from hardware devices, operating systems, databases, application software, user access logs, etc., analyzing the processing logs, and setting timely alarms for their abnormal conditions in several ways, such as sending out SMSs, emailing, and calling; (4) data desensitization at data collection terminals, including the desensitization of incremental data and real-time data, notably, the desensitization algorithms can be flexibly configured; (5) automatically analyzing multisource heterogeneous data and intelligently generating data mapping schemes greatly improves the efficiency of data integration; (6) analyzing the results of data collection and evaluating the data quality and the accuracy of field mapping; and (7) tracing the source of the data and providing graphical displays of the relationship between current fields and original fields, the logic of data calculation, and the results of intermediate calculations.

In addition, in the data collection process and subsequent data processing, this paper references some international medical information technology standards and specifications involving data collection (such as ICD-10, ICD-11, ICD-9, LOINC, SNOMED,UMLS, WHO ATC/DDD, CTCAE, etc.), management (such as HL7 CDA, CCD, openEHR, etc.), exchange and sharing (HL7 V2, HL7 V3, DICOM, etc.), privacy and security (IHE, HIPAA, ISO/TC 215 series, etc.), and other standards (HTML, XML, SOAP, HTTP, HIE, etc.). Hence, DSCDS can support personalized data configuration in different studies and include data from different sources commonly used in multicenter research.

### Governance and quality improvement of specific disease data

The collected data were standardized by using not only CDEs but also medical terminology and thesaurus, and they were all derived and extended from a variety of general medical information standards (such as ICD-10, LOINC, SNOMED, MeSH, ICD-9, CTCAE, CHPO, RxNorm, Classification and Code of Drugs in China's Social Insurance, Chinese Pharmacopoeia, etc.) [16, 17], as well as some international disease guidelines linked to specific diseases. Machine learning technologies were applied to intelligently infer metadata information, including data structures, data dictionary, and relationships between a primary key and foreign key. Natural language processing (NLP) [18], knowledge mapping, and artificial intelligence were used to clean, structuralize and standardize the data, as well as to manage master data, metadata and data quality control. The above processes were realized by adopting online analytical processing (OLAP) in a front-end processor system.

Enterprise master patient indexing (EMPI), uniquely identifying a patient, was used to integrate the data generated from multiple previous visits of patients, and it was the basis for building a patient-centered DSCDS and realizing the 360-degree panoramic view of a patient. A disease-specific clinical data repository was established using EMPI and standard data models, which realized data governance (including automatic data collection from different sources, data analysis and cleaning), data preprocessing (including data structuralization and standardization), indicator extraction based on logic and rules, rule mining, knowledge query, association analysis, image processing, anomaly detection and predictive analysis. Therefore, a unified and standardized mechanism of data governance and data quality improvement was created, ensuring data security, high data processing performance and cross-domain data transmission. DSCDS, which was established following this mechanism, could provide full-scale and real world data for clinical research on specific diseases. These data were also regular and of high quality.

### Application of storage and computing technology

Some storage and computing technologies with high performance, high reliability, and high scalability were applied to build a large DSCDS. The big data storage of DSCDS included both NoSQL databases (such as HBase, MongoDB, etc.) and relational databases (such as MySQL, etc.). The primary memory databases (such as Redis, Memcached, etc.) were introduced into the storage architecture of DSCDS to improve the speed of real-time calculation. The distributed computing frameworks of DSCDS included flow processing frameworks (such as Spark, Flink, etc.), batch processing frameworks (such as Hadoop, etc.), graph computing engines, data mining engines and some data analysis frameworks that contain artificial intelligence processing engines (such as TensorFlow and Pytorch, etc.) to meet the needs of different data analysis scenarios. In general, the storage system of DSCDS has the characteristics of a distributed structure and can handle the massive growth of data. Additionally, the storage system has the characteristics of a hierarchical structure, that is, it is composed of high-speed and low-speed storage; the high-speed storage ensures online real-time analysis, while the low-speed storage realizes offline batch processing. At the same time, the storage system of DSCDS also has complete data managing capability, and redundant data backup, synchronization, and isolation could be carried out based on it.

### Data application module design

**Fig. 2:**
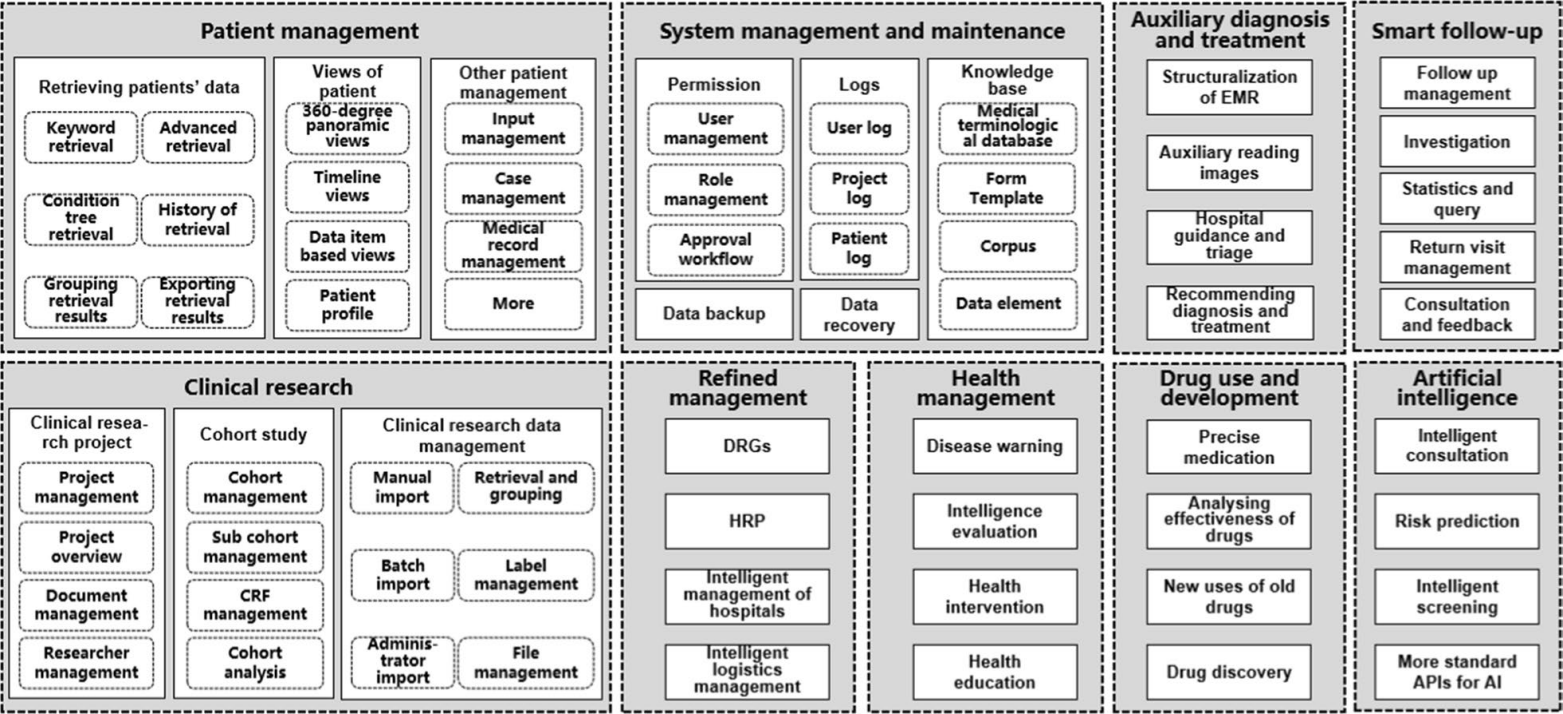
The design for the data application modules of DSCDS. *CRF* clinical report form, *DRGs* diagnosis-related groups, *HRP* hospital resource planning, *APIs* application program interfaces, *AI* artificial intelligence

Nine data application modules of DSCDS were designed, and they could provide many functionalities for medical services and clinical research on specific diseases, such as, for example, data search management, 360-degree panoramic view of patients, data collection management, data synchronization, permissions management, clinical research management, auxiliary diagnosis and treatment, intelligent follow-up, business intelligence (BI) analysis, health promotion, safe medication, and medical artificial intelligence (see Fig. 2 for details).

For example, the main purpose of building DSCDS was to support medical services and clinical research. The data application modules of DSCDS, designed concerning Fig. 2, met many needs of multiobjective big data studies on specific diseases, including clinical retrospective research, large-scale cohort studies, follow-up studies, related factor analysis, early prediction, risk prediction, prognosis prediction, etc. Therefore, the scientific research value of medical big data was truly excavated to assist the clinical research and decision-making of the specific disease.

## Results

### Basic information of the DSCDS for liver cirrhosis

**Fig. 3:**
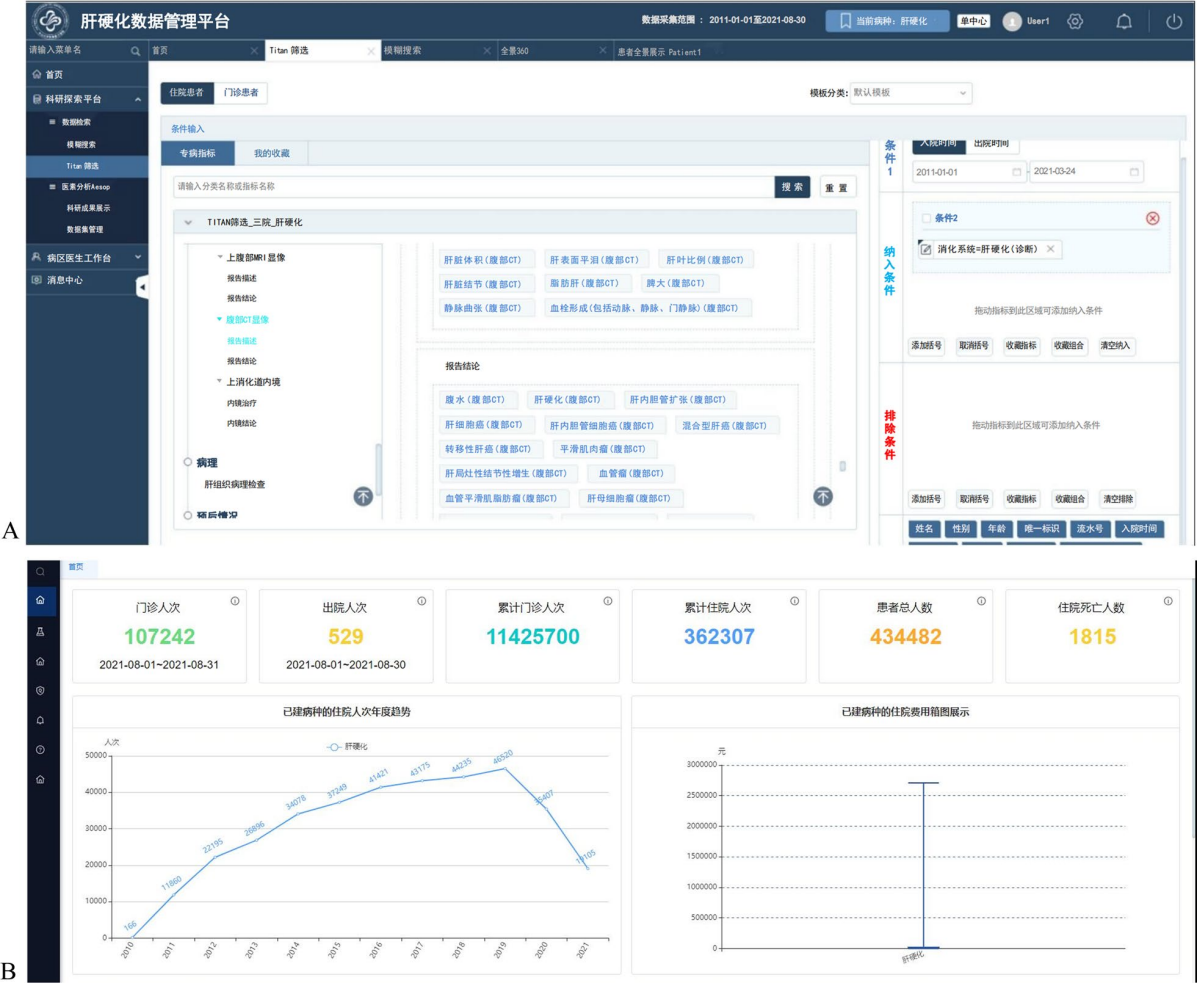
The interface of the DSCDS for liver cirrhosis. **A** Screening specific patients with liver cirrhosis according to inclusion criteria and exclusion criteria. **B** The number and visualization of patients and visits on the system homepage

In accordance with the above methods used in the design and implementation of DSCDS, a DSCDS for liver cirrhosis was built and implemented by relying on a 3A Grade hospital with the specialist advantage of gastroenterology in China. With the support of several departments and the construction companies of medical informatization in the hospital, this study analyzed the needs of the Department of Gastroenterology for DSCDS for liver cirrhosis. It combined the hospital business systems and the follow-up system to establish this DSCDS for liver cirrhosis. This DSCDS has aggregated medical data of 434,482 patients with liver cirrhosis and 11,788,007 (person-time) visits from January 1, 2011, to August 30, 2021, including 11,425,700 (person-time) outpatient visits and 362,307 (person-time) inpatient visits. Its dataset mainly includes multidimensional information such as conventional electronic medical records, pathological data, medical images, and clinical biological samples, with more than 700 CDEs. All patients with liver cirrhosis had complete electronic medical records, examination information and test information, 2,250 patients had pathological data, and 28,705 patients had medical images. The interface of this DSCDS for liver cirrhosis is shown in Fig. 3.

### Data security and use of the DSCDS for liver cirrhosis

To ensure data security and protect patient privacy, this DSCDS for liver cirrhosis is currently deployed in the intranet systems of the hospital and all users need to be authorized by administrators who have different permissions according to their professional titles to access this database system. It followed a data security and privacy protection system mentioned in the methods section to prevent data leakage and improper use. Its users were mainly gastroenterologists of this hospital and included doctors from other departments that had cooperative research with the gastroenterology department. Before using the data, users needed to submit a detailed research plan and obtain the approval of peer experts, the ethics committee, and the big data center in this hospital. At the same time, the private data of hospital departments, doctors and patients were completely desensitized. While using the data, the data source was traced to fully master the data usage path.

This DSCDS for liver cirrhosis can support data management, query, statistics and visualization, which are implemented by using EMPI (a unique identifier for each patient) and were required by many medical workers and researchers who have access to this DSCDS in this hospital for routine hospital management and clinical diagnosis and treatment of liver cirrhosis. However, some clinical studies on liver cirrhosis can be conducted based on this DSCDS. For example, according to specific research objectives, medical workers and researchers can filter the features of subjects with live cirrhosis after determining inclusion criteria, exclusion criteria and output indicators and online further describe and analyze the features of the subjects. Simultaneously, they can download the filtered data to carry out more in-depth research on data mining and artificial intelligence application for liver cirrhosis, such as large cohort studies, prognosis studies based on multimodal data, and postoperative follow-up studies. In addition, more algorithms and modules can access or be embedded in this DSCDS using a standard application programming interface (API), including artificial intelligence (such as machine learning, deep learning, etc.), data analysis algorithms and data mining algorithms, which can support data-driven medical services and clinical research on liver cirrhosis in the future.

### Application of the DSCDS for liver cirrhosis

The DSCDS for liver cirrhosis was in a dynamic process of development and improvement. Therefore, some of the data application modules in Fig. 2 were achieved, including all functions of patient management and system management and maintenance and some simple functions of clinical research, auxiliary diagnosis and treatment, and smart follow-up; however, refined management, health management, drug use and development, and artificial intelligence are still under design. Nonetheless, we are using this system to carry out some research. Currently, a series of retrospective and prospective studies are being conducted using this DSCDS for liver cirrhosis, including follow-up studies on the therapeutic effect and prognosis of gastroesophageal variceal hemorrhage, prognostic predictions of liver cirrhosis based on multimodal data, the potential correlation between medical history, genetic and imaging features and liver cirrhosis [19], and large cohort studies on the characteristics, influencing factors and complications of liver cirrhosis [20, 21]. We applied for a clinical trial to study the quantitative evaluation and reversible treatment of liver cirrhosis (registration number: ChiCTR1900023426).

At the same time, we are designing and implementing an intelligent follow-up management system for patients with liver cirrhosis based on this DSCDS to standardize the follow-up mechanism and process to promote follow-up compliance and patient satisfaction, reduce the risk of liver diseases, enhance the contact between be patients and medical workers, and improve the efficiency and success rate of follow-up. This system can be accessed from the mobile terminal and the web terminal. The mobile terminal was deployed on the WeChat account of this hospital, which is easy for users to operate. On the web side, nursing staff can manage patient accounts, maintain databases and timely statistical analysis data (http://157.255.121.64:3094/v3/#/login). We collected the baseline questionnaire data of nearly 300 patients with liver cirrhosis and gastroesophageal variceal hemorrhage. In the future, we will follow up with the patients and collect more data mainly about their lifestyle related to liver cirrhosis (such as diet, drinking, medication, exercise, etc.), preoperative situation of bowel preparation and colonoscopy, intraoperative situation of each sequential treatment, and postoperative situation of treatment and compliance. Written informed consent was obtained from each participant before all of the above studies began, and all studies were approved by the Ethics Committee of Sun Yat-sen University.

## Discussion

This paper describes the design and development of clinical database systems for specific diseases in Chinese hospitals and takes one DSCDS for liver cirrhosis as an example. This DSCDS is the first clinical database system to be designed to target only liver cirrhosis to facilitate the availability of hospital data in China. It was developed by adopting the methods described in the Methods section, which offers a common approach to collecting and aggregating medical data targeting a specific disease. These methods enable Chinese medical workers and clinical researchers to consider making full use of real world medical big data and incorporate information techniques into coresearch on a specific disease and improving the quality of medical service for the specific disease. In addition, this DSCDS is also an efficient novel medical informatization solution for effective hospital management and administering medical services centered on a specific disease. The uniqueness of this DSCDS lies in its new features, such as offering multimodal and comprehensive data, allowing services for medical services and clinical research on liver cirrhosis, and integrating various data application modules.

In contrast, most international clinical studies on a specific disease usually establish datasets to gather data for specific research purposes, including first selecting a target population based on inclusion and exclusion criteria, then determining data elements, and finally designing questionnaires and forms to collect the specific disease data from medical services and investigations. Therefore, most of those data are prospective and not real world data because they are often filtered and processed painstakingly, and the amount of those data is also relatively small. In contrast, DSCDS, constructed in Chinese hospitals, can collect prospective and retrospective data accumulated in the past, and most of these data are from medical systems in the real world with the advantage of having large amounts of data. Thus, DSCDS can be considered very helpful for carrying out more comprehensive and in-depth research on a specific disease.

The importance of the increasing availability of real world data in medical services and research on specific diseases in Chinese hospitals is being increasingly recognized, and several DSCDS practices have also been published in Chinese journals in recent years. These DSCDSs were designed and developed for specific diseases, and they could provide medical data and data application modules, which was different from general clinical database systems and CDRs. However, these practices did not take clear, uniform and standard approaches to designing and developing these DSCDSs. Therefore, these DSCDSs were difficult to use widely and be referenced by other developments of DSCDSs; however, they were not built for liver cirrhosis. In this paper, the approaches adopted in designing and implementing the DSCDS for liver cirrhosis were systematic and comprehensive and could offer relatively uniform and standard approaches to the design and development of DSCDS in China and even in other countries. These approaches ensured that all methods, technologies, and procedures were flexibly used according to different construction conditions of DSCDS. Further, these approaches were highly applicable to the construction of different DSCDSs, provided a clear framework for the design and development of DSCDSs, and facilitated their replicability. Therefore, the DSCDS built by these approaches can maximize the application potential of specific disease data to increase medical data availability in Chinese hospitals.

The development of the DSCDS for liver cirrhosis had several limitations. First, although one of the core advantages of the DSCDS for liver cirrhosis is to collect and store medical data from hospital business systems to ensure more comprehensive and multimodal data. However, as previously reported on the developments of CDRs, the construction of DSCDS is also a time-consuming and labor-intensive process, which requires collaboration with medical workers, hospital departments and HIT companies that construct hospital business systems [1, 2]. Therefore, the construction of this DSCDS has not strictly adopted all the common methods and techniques described in the Methods section, although it applied the same design and development framework. For example, this DSCDS has not yet accessed more multimodal data, such as omics data (genomic data, proteomic data and metabolomic data) and clinical biological samples; and pathological data and medical images have not yet been completely collected. In addition, its data application modules have not been achieved comprehensively and still need further design, implementation, testing and improvement. Second, despite the systematic design and implementation steps used in the development of this DSCDS, different developments of DSCDSs using the same methods would need to solve different problems arising from various specific diseases, hospitals and medical workers’ and researchers’ needs. Therefore, in the whole process of design and development of DSCDS, it is necessary to flexibly design solutions according to appropriate characteristics of various specific diseases, technical experience (from different database developers, medical workers and researchers) and actual considerations of the whole hospital [22]. The approaches of developing DSCDS also need to be finely adjusted to adapt to the practical conditions in actual practices and even develop with the progress of new technologies. Finally, this DSCDS for liver cirrhosis is still preliminarily developed and applied, and its application in medical services and clinical research is not extensive and in-depth enough.

In the future, more research will be carried out to address the limitations discussed above, provide insights into how this DSCDS for liver cirrhosis can be further improved in terms of usability, and enable this DSCDS to better meet the need for medical services and clinical research on liver cirrhosis. For instance, more functional applications will be expanded on, including smart medical scenarios based on multimodal medical big data fusion, such as clinical decision support and intelligent auxiliary diagnosis for liver cirrhosis and hepatocellular carcinoma. Furthermore, further research should include assessment of the effectiveness of this DSCDS, including timely evaluation and precise treatment of patients and improving the efficiency of clinical research and the quality of medical service. In addition, more standard systems related to the development of DSCDS will be built and adopted to promote the exploratory application of DSCDS in more scenarios, such as tiered medical services, telemedicine, two-way referral and intelligent auxiliary diagnosis.

Beyond these, DSCDS aims to provide a practical approach to increasing the availability of real world data in Chinese hospitals, which can promote the digitalization of medical services and clinical research on specific diseases, and to some extent, can improve the development of medical informatization of hospitals in China. To achieve this long-term goal, this DSCDS for liver cirrhosis, as a typical case, will be scaled up and extended to other healthcare institutions to realize a digital medicine network for liver diseases, from which a larger multicenter DSCDS can be developed to support smart healthcare management, decision support, and clinical research for liver diseases. The multicenter DSCDS will collect multicenter health care datasets and support the establishment of regional medical centers, medical consortia, medical quality control centers and key laboratories for liver diseases, which will further promote the construction of a smart medical system from a hospital to a region. This smart medical system has many characteristics, such as resource integration, data interconnection and information sharing; and it can make the clinical research, diagnosis and treatment of liver diseases more intelligent, automated, standardized, and networked. DSCDSs, as a solid foundation of the above, are important trends to improve the level of regional medical diagnosis, and treatment of liver diseases in the future.

## Conclusion

High-quality data are the key basis of the development of digital medicine. The development of DSCDS, a solution enhancing the availability of hospital data, is very suitable for the current development of hospital medical informatization in China. In this study, the DSCDS for liver cirrhosis, with the intent to combine liver cirrhosis data in the real world, provides high-quality data for clinical research, clinical decision-making, and improvement of medical service quality of liver cirrhosis and will further drive the development of smart medicine centered on liver disease. In China, DSCDS is still in the early stages of development. The design and development of this DSCDS formed a clear, standard and systematic approach involving data collection, data aggregation, data storage, data usage, and the application to data-driven medical service and clinical research of a specific disease. All these studies provided a reference approach (methods, techniques, experiences, relevant standards, etc.) to more hospitals to construct and apply other DSCDSs to improve medical services and clinical research management for specific diseases in China and can even be used extensively in other countries.

### Supplementary Information

Below is the link to the electronic supplementary material.

Supplementary file1 (DOCX 28 kb)

## Data Availability

The data that support the design and development of the disease-specific clinical database system for liver cirrhosis in this study are available from The Third Affiliated Hospital of Sun Yat-sen University. Restrictions apply to the availability of the data, which were used under license for this study. The data are not publicly available due to security, privacy, or ethical restrictions.
