# Supplementary material for: Design and development of a disease-specific clinical database system to increase the availability of hospital data in China
Source: Health Inf Sci Syst. 2023 Jan 30;11(1):11. doi: 10.1007/s13755-023-00211-4 (PMC9886700; doi:10.1007/s13755-023-00211-4)
Supplement: Supplementary file 1 — Supplementary file1 (DOCX 28 kb) [file 13755_2023_211_MOESM1_ESM.docx]

**Appendix** The common data elements (CDEs) of CSDDS

| **First classification** | **Secondary classification** | **The PDEs** | **The SDEs ( for example liver cirrhosis)** |
| --- | --- | --- | --- |
| Clinical visit information | Personal identification | Patient ID; health card No. | - |
|  | Clinical visit identification | Outpatient No.; inpatient No.; medical record No.; type of clinical visit | - |
|  | Clinical visit records | Date and time of clinical visit; clinical visit departments; clinical visit process | More data elements about the clinical visit records of a specific disease |
| Demographic information | Personal information | Name; sex; age; date of birth; ID card type; ID card number; ethnicity; occupation; nationality; marital status; education level; work unit; medical insurance type; phone number; ABO blood type; Rh blood type | - |
|  | Address | Address type; address-province (autonomous region, municipality directly under the central government); address-city (state, region); address-county (district); address-township (town, Sub-district office); address-village (street, road, lane, etc.); address-door number; postal code | - |
|  | Contact information | Contact name; relationship with the contact; phone number of the contact | - |
| Health history | Past medical history | Have previous disease; type of previous disease; name of previous disease; date of previous disease; duration of previous disease; have received drug therapy; drug name | More data elements about medical history linked to a specific disease (e.g., details of past hepatic diseases, such as hepatitis B, hepatitis C, hepatitis A, hepatitis D, hepatitis E, intrahepatic cholelithiasis, liver cirrhosis, alcoholic liver disease, fatty liver, liver cancer and so on) |
|  | Surgical history | Have had surgery; name of surgery; date of surgery; surgical site; surgical procedure | More data elements about surgical history linked to a specific disease (have had liver surgery; name of disease diagnosed before liver surgery; procedure of liver surgery, etc.) |
|  | Smoking history | Smoking or not; years of smoking ; number of cigarettes smoked daily; quit smoking or not; years of quitting smoking | - |
|  | History of alcohol consumption | Drinking or not; daily alcohol consumption; years of drinking; quit drinking or not; years of quitting drinking | - |
|  | Reproductive history (Female) | Have given birth; number of sons ; number of daughters | More data elements about reproductive history linked to a specific disease |
|  | Menstrual history (Female) | Date of last menstruation; age of menarche; menstrual cycle; age of menopause | More data elements about menstrual history linked to a specific disease |
|  | Familial disease history | Have familial disease history; relationship with the patient; name of the familial disease; onset age of the patient with familial disease; family members have did gene tests; gene test result of the family members | More data elements about family history linked to a specific disease (more details of family members with liver cirrhosis or cancer, including diagnosis, medical assessment and intervention, etc.) |
|  | Allergy history | Have the history of allergies; name of the allergen | More data elements about allergy history linked to a specific disease (have be allergic to hepatoprotectants; name of that hepatoprotectants; description of this allergic reaction, etc.) |
|  | More life histories linked to a specific disease (e.g., exposure to pathogenic factors, blood transfusion, drug abuse, tattoo) | - | More data elements about life histories linked to a specific disease (have eaten moldy food; have be exposed to water with schistosome; have kept cats or dogs, have eaten sashimi, have had antiviral therapy of hepatitis, time of starting antiviral therapy of hepatitis, etc.) |
| Chief complaint and symptom | Chief Complaint | Chief complaint; date of onset | - |
|  | Symptoms (Symptoms vary widely among specific diseases, many of them are common symptoms of specific diseases, such as weight change, abdominal pain, fatigue, fever, anorexia, abdominal distention, diarrhea, hemoptysis, hematemesis, melena, anemia, abdominal mass, etc.) | Name of symptom; date of first onset of the symptom; duration of the symptom | More data elements about symptoms linked to a specific disease (have symptoms related to liver disease, such as spider nevus, edema of both lower limbs, abdominal mass, acholic stool, etc., including names and severity of these symptoms) |
| Physical examination | General examination | Date of physical examination; height; weight; body mass index | - |
|  | Vital signs | Date and time of measurement; body temperature; heart rate; respiratory rate; diastolic blood pressure; systolic blood pressure | - |
|  | Score | Date of score; KPS score; ECOG score | - |
|  | More physical examinations linked to a specific disease (e.g., state of consciousness, nutritional status, abdominal tenderness, perianal pathological changes) | - | More data elements about physical examination linked to a specific disease (e.g., have hepatic encephalopathy, and its details) |
| Laboratory examination | Blood routine examination | Time of the examination; label of abnormality; reference range of the examination result; white blood cell count; monocyte count; red blood cell count; absolute value of neutrophil; absolute value of lymphocyte; absolute value of eosinophil; absolute value of basophil; neutrophil percentage; lymphocyte percentage; basophil percentage; eosinophil percentage; monocyte percentage; platelet count; hemoglobin detection value; mean corpuscular volume; mean corpuscular hemoglobin content; mean corpuscular hemoglobin concentration; red blood cell volume distribution width; platelet distribution width; mean platelet volume; platelet-large ratio; hematocrit; plateletcrit | - |
|  | Routine urine examination | Time of the examination; label of abnormality; reference range of the examination result; count of leukocytes in urine; specific gravity of urine; result of qualitative test of urine protein; count of erythrocyte in urine; result of urine occult blood test; result of qualitative test of urine glucose; result of qualitative test of urine ketone; urine pH; result of qualitative test of urine bilirubin; result of urobilinogen test | - |
|  | Blood biochemical examination | Time of the examination; label of abnormality; reference range of the examination result; albumin concentration; total protein detection value; globulin detection value; albumin/globulin ratio; alanine aminotransferase detection value; aspartate aminotransferase detection value; blood cholinesterase activity; lactate dehydrogenase detection value; γ-glutamyl transferase detection value; glutamate dehydrogenase detection value; serum total bilirubin detection value; conjugated bilirubin detection value; blood glucose detection value ; triglyceride detection value; very low density lipoprotein cholesterol detection value; serum low density lipoprotein cholesterol detection value; serum high density lipoprotein cholesterol detection value; total cholesterol detection value; blood urea nitrogen detection value; blood creatinine detection value; uric acid detection value; blood urea nitrogen/creatinine ratio; blood potassium concentration; blood sodium concentration; blood calcium concentration; blood magnesium concentration; inorganic phosphorus concentration; creatine kinase detection value; creatine kinase isoenzyme detection value; ischemia modified albumin detection value; alkaline phosphatase concentration | More data elements about blood biochemical examination linked to a specific disease (transthyretin concentration; blood chlorine concentration; blood ammonia concentration; phospholipase A2 detection value, ect.) |
|  | Hepatitis B test | Time of the test; label of abnormality**;** reference range of the test result; hepatitis B virus surface antigen test results; hepatitis B virus surface antibody test results; hepatitis B virus e antigen test results; hepatitis B virus e antibody test results; hepatitis B virus core antibody test results | More data elements about hepatitis B test linked to a specific disease (amount of serum HBV DNA, ect.) |
|  | More infectious disease examinations linked to a specific disease (e.g., hepatitis A, hepatitis C, hepatitis D, hepatitis E, syphilis) | - | More data elements about infectious diseases examination linked to a specific disease (hepatitis A virus surface antigen test results, hepatitis C virus surface antigen test results, amount of serum HCV DNA, hepatitis D virus surface antigen test results, hepatitis E virus surface antigen test results, ect.) |
|  | Feces routine examination | Time of the examination; label of abnormality; reference range of the examination result; fecal occult blood test results; white blood cell count in feces; red blood cell count in feces | - |
|  | Routine examination of hemorrhage and coagulation | Time of the examination; label of abnormality; reference range of the examination result; Plasma D-dimer detection value; prothrombin time detection value; activated partial thrombin time detection value; international normalized ratio of prothrombin time detection value; thrombin time detection value; fibrinogen detection value; fibrinogen degradation products detection value | - |
|  | More laboratory tests linked to a specific disease (e.g., tumor markers detection, sex hormone detection, disease-specific antigens detection, gene test, liver function test, determination of liver fibrosis, kidney function tests) | - | More data elements about laboratory tests linked to a specific disease (e.g., determination of liver fibrosis, including hyaluronic acid, fibronectin, type IV collagen, laminin; tumor markers detection, including alpha fetoprotein, carcinoembryonic antigen CEA, CA125, ferritin iron) |
| Clinical assistant examination | Imaging examination (including X-ray, CT, magnetic resonance, ultrasound, nuclear medicine, etc.) | Date of image examination; type of image examination; site of image examination; name of examination; location of lesion; number of lesion; size of lesion; diameter of lesion; type of lesion | More data elements about imaging examination linked to a specific disease (have pulmonary emphysema; have secondary tumor; have cardiac enlargement; have the widened mediastinal; have pleural effusion; have ascites; have liver cirrhosis; have splenomegaly; have varicose veins; have fatty liver; upper-down diameter of left liver; left-right diameter of left liver; anterior-posterior diameter of left liver; oblique diameter of right liver; thickness of right liver; diameter of portal vein; have intrahepatic bile duct dilatation; have hepatocellular carcinoma; have intrahepatic cholangiocarcinoma; have mixed cholangiocarcinoma; have metastatic liver cancer; have leiomyosarcoma; have hepatic focal nodular hyperplasia; have hemangioma; have angiomyolipoma; have hepatoblastoma; have hilar cholangiocarcinoma; have choledochal carcinoma; have hepatic adenoma; have hepatic abscess; have intrahepatic calcification; have intrahepatic cholelithiasis; hepatic shrinkage; normal hepatic morphology; smooth surface of liver; hyperechogenicity of liver parenchyma; have the blunted hepatic margin; normal proportion of hepatic lobe; value of hepatic CT plain scan; value of hepatic CT scan in arterial phrase, ect.) |
|  | More clinical assistant examinations linked to a specific disease (e.g., immunohistochemistry, pathological examination, pathological biopsy, endoscopy) | Date of examination; type of examination; location of examination; name of examination; result of examination | More data elements about clinical assistant examination linked to a specific disease (e.g., upper gastrointestinal endoscopy, including have esophageal varices (EV), have EV with active bleeding, site of EV, morphology of EV, color of EV, have red EV, have EV with esophagitis, grading of EV, gastric varices (GV), have GV with active bleeding, site of GV, morphology of GV, color of GV, have red GV, have GV with fundus gastritis, grading of GV, have gastritis, have gastric ulcer, have duodenal ulcer, have duodenitis, tumor invading stomach or duodenum, pathological examination No.; pathological examination, including grading of hepatitis, grading of liver fibrosis, detection of hepatic elasticity including liver stiffness, liver attenuation coefficient, spleen stiffness, etc.) |
| Diagnosis | Clinical Diagnosis | Type of diagnosis; name of disease diagnosed; code of disease diagnosed; date of diagnosis; condition on arrival at hospital | - |
|  | Medical diagnosis on death | Name of medical diagnosis on death; code of medical diagnosis on death; direct cause of death; code of direct cause of death; date and time of death | - |
|  | More medical diagnoses linked to a specific disease (e.g., stage of disease, first diagnosis of tumor, complication) | - | Medical data elements about diagnosis linked to a specific disease (grading of hepatitis, grading of liver fibrosis, staging of liver cirrhosis, ect.) |
|  | More comorbidities linked to a specific disease (e.g., malignant tumor, kidney disease, diabetes, hypertension, heart disease, cerebrovascular disease) | - | More data elements about comorbidities linked to a specific disease (details of comorbidities about liver cirrhosis, such as hepatic encephalopathy, hepatorenal syndrome, primary hepatic carcinoma, hepatopulmonary syndrome, portal vein thrombosis, primary peritonitis, upper gastrointestinal bleeding, etc.) |
| Medical assessment | Grouping of risk | Criteria of grouping of risk; result of grouping of risk; date of grouping of risk | - |
|  | Relapse | Have relapse; form of relapse; date of diagnosis of relapse | - |
|  | More medical evaluation linked to a specific disease (e.g., tumor metastases, etc.) | - | More data elements about medical assessment linked to a specific disease (metastasis of hepatocellular carcinoma; site of metastasis; diagnosis date of metastasis, ect.) |
| Medical planning and intervention | Hospitalization information | Source of inpatient**;** times of hospitalization; date of admission in hospital; date of discharge from hospital; department admitted in hospital; department discharged from hospital; actual days of hospitalization; way of discharge; main diagnosis on admission in hospital; other diagnosis on admission; treatment; main discharge diagnosis; other discharge diagnosis; treatment process of this hospitalization | More data elements about hospitalization linked to a specific disease |
|  | Drug therapy | Classification of medication; drug name; purpose of medication; single dose administration of drug; drug dosage units ; frequency of drug use; route of administration; date and time of starting administration; end date of administration; evaluation of drug curative effect; evaluation date of drug curative effect; way of evaluation of drug curative effect; description of adverse drug reaction; count of medication cycles | More data elements about drug therapy linked to a specific disease (e.g., anti hepatitis virus drug; drug for treating symptoms of liver cirrhosis, such as treating ascites, protecting liver, protecting gastric mucosa, have anti liver fibrosis effects, etc.; and hemostatic, antidiarrheic, cholagogue, somatostatin, proton pump inhibitors of gastric parietal cells and H2 receptor blockers for gastric parietal cells, etc.) |
|  | Clinical trials | Have participated in clinical trial; type of treatment in clinical trial; status of clinical trial; clinical trial number; name of clinical trial; date of participation in clinical trial; date of withdrawal from clinical trial; reason for clinical trial suspension; overall treatment effect in clinical trial; evaluation date of treatment effect; way of evaluation of treatment effects | More data elements about clinical trials linked to a specific disease (e.g., details of adverse events, including have adverse events, occurrence of adverse event, name of adverse event, the adverse event is related to the clinical trial, severity of adverse event, start time of adverse event, end time of adverse event, adverse event outcome, whether to withdraw from the clinical trial, etc.) |
|  | Surgery | Surgical approach; surgery name; surgery code; type of surgery; surgical grade; surgery date; surgical duration ; volume of intraoperative bleeding; have intraoperative blood transfusion; component of intraoperative blood transfusion; volume of intraoperative blood transfusion; have lymph node dissection; scope of regional lymph node dissection; have intraoperative complications; description of intraoperative complications; treatment of intraoperative complications; have sample submitted for examination; name of sample submitted for examination; date of surgical resection of the primary site | More data elements about surgery linked to a specific disease (name of anesthetic; dosage of anesthetic; injection site of anesthetic, ect.) |
|  | Postoperative recovery | Have postoperative complications; date of occurrence of postoperative complication; name of postoperative complication name; treatment of postoperative complication; grade of incision healing; have postoperative drainage; location of drainage; duration of drainage; postoperative hospital stay | More data elements about postoperative recovery linked to a specific disease (have adverse event; name of adverse event; effect of surgery; evaluation date of surgery; way of evaluation of surgery, etc.) |
|  | More operations linked to a specific disease (e.g., needle biopsy for malignant tumor) | - | More data elements about operation linked to a specific disease (site of biopsy; guidance mode; puncture approach; number of punctures; have complications; description of complications; have repeated puncture, etc.) |
|  | More treatments linked to a specific disease (e.g., antiviral therapy, radiotherapy, and symptomatic treatment and endoscopic therapy) | Name of treatment; beginning date of treatment; end date of treatment; description of adverse effect; evaluation of treatment effect; evaluation date of treatment effect; way of evaluation of treatment effect | More data elements about treatment linked to a specific disease (e.g., endoscopic therapy of upper digestive tract, including have endoscopic variceal ligation, number of ferrules in ligation, use tissue glue, amount of tissue glue used, use hardener) |
|  | Treatment evaluation | Treatment evaluation; date of treatment evaluation; way of treatment evaluation; overall treatment evaluation | - |
|  | Follow-up information | Follow-up or not; classification of follow-up status; way of follow-up; date of follow-up; number of follow-up; date of withdrawal of follow-up management; reason for withdrawal of follow-up management | More data elements about follow-up information linked to a specific disease (e.g. symptoms, treatment compliance, recurrence, metastasis, revisit, retreatment, etc. e.g., have some symptoms, including fever, abdominal pain, anorexia, fatigue, weight loss, weight gain, diarrhea, abdominal distension, hemoptysis, hematemesis, black stool, disturbance of consciousness and so on; treatment compliance; forget to take medicine; take another medicine or interruption; reasons for taking another medicine or interruption; recurrence; metastasis; type of revisit; type of retreatment, etc.) |
| Clinical biological samples | Serum | Time of collection serum; time of storing serum in the laboratory; sub-package amount; placement location; laboratory number; responsible person | - |
|  | Feces | Time of collection feces; time of storing feces in the laboratory; sub-package amount; placement location; laboratory number; responsible person | - |
|  | More clinical biological samples linked to a specific disease (e.g. tissue, saliva, urine, bone marrow) | Time of collection samples; time of storing samples in the laboratory; sub-package amount; placement location; laboratory number; responsible person | More data elements about clinical biological samples linked to a specific disease (size of tissue; volume of tissue; color of tissue, etc.) |
| Medical expenditure | Medical expenditure | Date of payment; outpatient fee or not; amount of outpatient fee; payment way of outpatient fee; inpatient fee or not; amount of inpatient fee; payment way of inpatient fee; amount of fees paid by individual | - |
| Medical institution | Institution identification | Institution name; organization code | - |
